# Clinical significance of E2F1 protein expression in non-small cell lung cancer

**DOI:** 10.1186/2162-3619-1-18

**Published:** 2012-07-20

**Authors:** Jung-Jyh Hung, Chung-Tsen Hsueh, Kuan-Hua Chen, Wen-Hu Hsu, Yu-Chung Wu

**Affiliations:** 1 Division of Thoracic Surgery, Department of Surgery, Taipei Veterans General Hospital, Taipei, 112, Taiwan; 2 School of Medicine, National Yang-Ming University, Taipei, 112, Taiwan; 3 Division of Medical Oncology and Hematology, Loma Linda University, Loma Linda, California, USA; 4 Department of Health Risk Management, School of Public Health, China Medical University, Taichung, Taiwan

**Keywords:** E2F1, Protein expression, Non-small cell lung cancer, Survival, Freedom from recurrence

## Abstract

**Background:**

The transcription factor E2F1 has been implicated in cell cycle control and DNA damage response. Paradoxically, E2F1 can promote apoptosis and function as tumor suppressor. In non-small cell lung cancer (NSCLC), there are conflicting data for clinical significance of E2F1 expression. In this study, we investigated the protein expression of E2F1 in patients with stage I-III NSCLC, and its correlation with clinical outcome.

**Results:**

56 paired adjacent non-tumor/tumor matched samples were prospectively obtained from patients undergoing surgery for stage I-III NSCLC at Taipei Veterans General Hospital. The protein expression of E2F1 was determined by Western blot analysis. The levels of E2F1 protein were significantly higher in tumor samples than in non-tumor lung specimens (*P* = 0.008). Overexpression of E2F1 was defined as a more than 2-fold expression in the tumorous sample compared with the corresponding nontumorous one, and was noted in 21 patients (37.5%). There was no significant difference in overall survival (*P* = 0.44) or probability of freedom from recurrence (*P* = 0.378) between patients with E2F1 overexpression vs. non-overexpressors. Additionally, there was no significant association between E2F1 overexpression and any clinicopathologic parameter such as histological type, stage, or angiolymphatic invasion of tumor.

**Conclusion:**

E2F1 protein is frequently overexpressed in NSCLC. There is no correlation between E2F1 protein expression and clinical outcome such as survival and freedom from progression.

## Background

Lung cancer is the leading cause of cancer-related death globally, and non-small cell lung cancer (NSCLC) accounts for more than 80% of all lung cancers. Despite of advances in biomedical science, surgery remains the mainstay of treatment for early-stage NSCLC, and the result of surgical treatment alone is unsatisfactory. Therefore, there is an urgent need to identify biomarkers for prognostication of patients’ outcome after surgery. Prognostic markers can be used to select patients with increased risk of recurrence who may require more frequent surveillance and derive more benefit from adjuvant treatment. Many prognostic markers have been investigated in NSCLC such as mutation status of KRAS and p53, and expression of excision repair cross-complementation group 1 and ribonucleotide reductase subunit M1 [1-3].

E2F1 belongs to the E2F family of transcription factors, and plays an important role in cell cycle progression and apoptosis [4]. Besides cell cycle regulation, E2F1 participates in DNA damage response and functions as a checkpoint control [5]. Increased E2F1 protein level associated with DNA damage and repair is observed in NSCLC cells after exposure to cisplatin [6]. Furthermore, E2F1 can induce apoptosis by both a p53-independent mechanism (mediated by p73) and a p53-dependent mechanism in cancer cell lines [7,8]. Development of NSCLC and lymphoma has been observed in mice lacking E2F1, indicating the role of tumor suppressor for E2F1 [9,10].

Dysregulation of E2F1 is frequently seen in cancers. It seems that E2F1 plays a dual role in terms of promoting tumor growth and inducing apoptosis. For example, E2F1 overexpression is associated with better clinical outcomes in diffuse large B-cell lymphoma, urinary bladder cancer, tongue cancer, gastric cancer and esophageal adenocarcinoma [11-15]. Whereas in breast and thyroid neoplasms, increased E2F1 expression is associate with higher proliferation index and more aggressive profile [16,17]. For NSCLC, conflicting data about the prognostic value of E2F1 expression exist in patients undergoing surgery for stage I-III disease. Volm et al. have examined E2F1 expression in 96 patients with squamous-cell lung carcinoma by immunohistochemistry (IHC), and found no correlation between the E2F1 expression and survival [18]. Huang and others have studied E2F1 gene expression by quantitative RT-PCR in 127 patients with NSCLC, and found adverse clinical outcomes in patients with E2F1 overexpression [19]. These findings imply E2F1 could be a double-edged sword, and more study is needed to explore its role as a prognostic marker in NSCLC. Herein, we investigated the clinical significance of E2F1 protein expression by Western blot analysis in a cohort of patients with stage I-III NSCLC undergoing surgical resection.

## Results and discussion

After surgery, the patients were followed regularly with physical examination and imaging study. The mean follow-up duration for all the 56 patients was 54.6 ± 34.1 months. The characteristics of these patients are listed in Table 1. There were 4 patients undergoing adjuvant chemotherapy, 7 undergoing adjuvant radiotherapy, and 2 receiving adjuvant chemoradiation therapy. At the last follow-up, 26 (46.4%) were free of tumor recurrence, 27 (48.2%) developed recurrence, and 3 (5.4%) patients had unknown recurrence status.

**Table 1:** Characteristics of 56 patients with non-small cell lung cancer and the relationship between E2F1 protein expression and clinicopathological variables

**Variables**	**Total (n = 56)**	**E2F1 protein expression**		
		**Low (n = 35)**	**High (n = 21)**	** *P* value**
Age at operation, years	65.9 ± 11.1	66.8 ± 10.0	64.6 ± 12.8	NS
Gender				
Male	41 (73.2)	27 (77.1)	14 (66.7)	NS
Female	15 (26.8)	8 (22.9)	7 (33.3)	
Smoking index, pack-years	25.1 ± 32.0	29.6 ± 36.1	17.9 ± 23.1	NS
Tumor size, cm	4.0 ± 1.6	4.0 ± 1.7	3.8 ± 1.5	NS
Histological type				
Squamous cell carcinoma	20 (35.7)	14 (40.0)	6 (28.6)	NS
Adenocarcinoma	33 (58.9)	20 (57.1)	13 (61.9)	
Others	3 (5.4)	1 (2.9)	2 (9.5)	
T descriptor				
T1	7 (12.5)	3 (8.6)	4 (19.0)	NS
T2	38 (67.9)	25 (71.4)	13 (61.9)	
T3	4 (7.1)	1 (2.9)	3 (14.3)	
T4	7 (12.5)	6 (17.1)	1 (4.8)	
N descriptor				
N0	33 (58.9)	20 (57.1)	10 (61.9)	NS
N1	10 (17.9)	8 (22.9)	4 (9.5)	
N2	13 (23.2)	7 (20.0)	7 (28.6)	
Stage				
I	26 (46.4)	16 (45.7)	10 (47.6)	NS
II	10 (17.9)	6 (17.1)	4 (19.0)	
III	20 (35.7)	13 (37.2)	7 (33.4)	
Angiolymphatic invasion				
Absent	38 (67.9)	23 (67.6)	15 (75.0)	NS
Present	16 (28.5)	11 (32.4)	5 (25.0)	
Unknown	2 (3.6)			
Histological grade				
Well differentiated	4 (7.1)	3 (8.6)	1 (5.3)	NS
Moderately differentiated	36 (64.3)	23 (65.7)	13 (68.4)	
Poorly differentiated	14 (25.0)	9 (25.7)	5 (26.3)	
Unknown	2 (3.6)			

*NS* not significant.

Continuous variables are expressed as mean ± SD; categorical variables are expressed as N (%).

**Figure 1:**
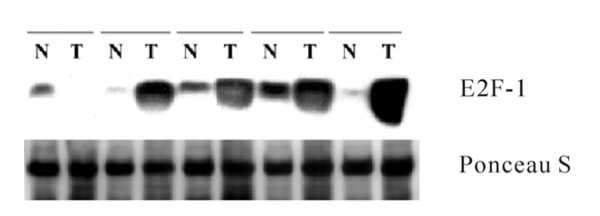
**Immunoblotting was performed on representative five pairs of non- tumor (N) lung tissues and tumorous (T) specimens.** Ponceau-S staining was used as control.**Immunoblotting was performed on representative five pairs of non- tumor (N) lung tissues and tumorous (T) specimens.** Ponceau-S staining was used as control.

A total of 56 pairs of matched fresh frozen tumor specimens and non-tumor normal lung tissues were used for Western blot analysis. The representative results of Western immunoblotting in 5 pairs of specimens were shown in Figure 1. As shown in Table 2, the mean E2F1 protein expression in tumors and non-tumor lung tissues were 0.33 ± 0.04 and 0.19 ± 0.02, respectively. The mean E2F1 protein expression in normal lung tissues was. E2F1 protein expression was significantly higher in tumor specimens than in normal lung tissues (paired *t*-test, *P* = 0.008).

**Table 2:** E2F1 protein expression in tumor and non-tumor specimens

	**Number**	Mean^*^	**Standard deviation**	**P value**
Tumor	56	0.33	0.04	0.008
Non-tumor	56	0.09	0.02	

^*^The membranes were exposed to NEN Renaissance x-ray film. The linear-range signal intensity of each specific band on the fluorogram is quantitated by a densitometric scanning system and comparison of proteins of interest is performed after normalization to the densitometric scanning of the Ponceau S staining. The control value of Ponceau S was assigned an arbitrary unit of 1, and the expression of each protein was denoted as arbitrary densitometry units (ADU) relative to the corresponding value of Ponceau S stain.

**Figure 2:**
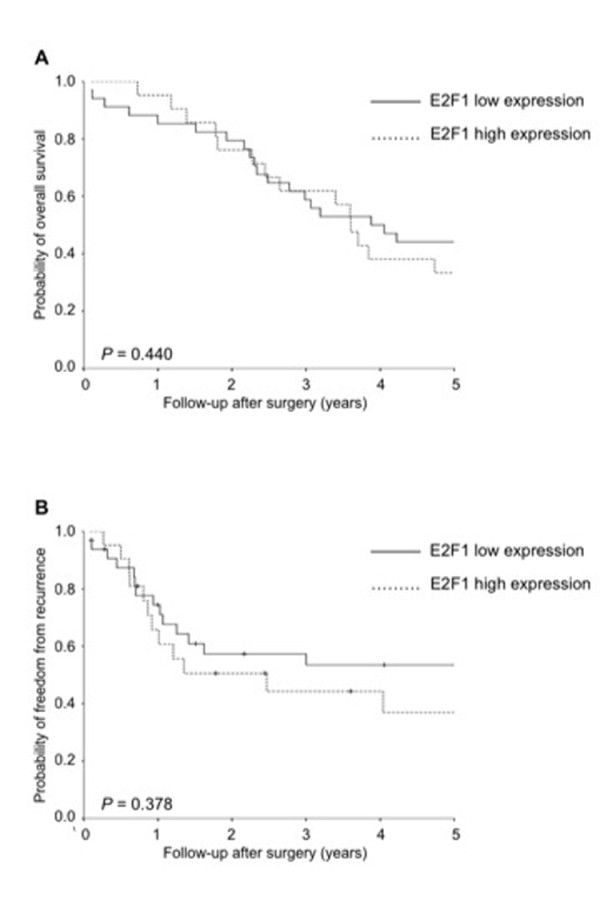
**(A) Cumulative probability of overall survival stratified by E2F1 protein low and high expression.** (**B**) Cumulative probability of freedom from recurrence stratified by E2F1 protein low and high expression. (log-rank test).

Overexpression of E2F1, defined as a more than 2-fold expression in the tumorous sample compared with the paired non-tumor tissue, was noted in 21 (37.5%) patients. As shown in Figure 2, the overall survival and probability of freedom from recurrence were not significantly different between patients with and without E2F1 overexpression (P = 0.440 and 0.378, respectively). We also analyzed the prognostic value of E2F1 expression in patients with NSCLC stratified by different stages. For patients with stage I NSCLC, the overall survival and probability of freedom from recurrence were not significantly different between patients with and without E2F1 overexpression (*P* = 0.385 and 0.306, respectively). For patients with stage II/III NSCLC, the overall survival and probability of freedom from recurrence were not significantly different between patients with and without E2F1 overexpression (*P* = 0.782 and 0.874, respectively). We further investigated the relationship between clinicopathological variables and E2F1 protein expression. There was no significant association between histological type and E2F1 expression (*P* = 0.906). Additionally, there was no relationship between E2F1 protein expression and other variables such as age, gender, smoking index, tumor size, T descriptor, N descriptor, stage, angiolymphatic invasion of tumor, and histological grade (Table 1).

We have summarized all 4 studies investigating clinical significance of E2F1 expression in patients undergoing surgical resection for stage I, II and III NSCLC (Table 3). Volm et al. used IHC to study E2F1 protein expression in 96 patients with squamous cell carcinoma of lung, and found no significant difference in survival between patients with E2F1 overexpression (IHC +++) and E2F1 non-overexpressors (IHC −/+/++)[18]. Gorgoulis and colleagues used IHC in 77 patients, and found significantly decreased survival in patients with above-median E2F1 protein immunoreactivity [20]. Huang et al. reported that increased E2F1 RNA expression was correlated with decreased survival in 127 patients [19]. However, for 57 patients with stage I NSCLC, they did not observe any correlation between survival and E2F1 RNA expression.

**Table 3:** Studies on clinical significance of E2F1 expression in NSCLC

**Study/year**^1^	**Patient number**	**Adenocarcinoma/SCC**	**Assay used**	**Incidence of E2F1 overexpression**	**Survival difference**
Volm/1998	96	0/96	IHC	24%	No
Gorgoulis/2002	77	37/37	IHC	49%	Yes
Huang/2007	127	63/58	Quantitative RT-PCR	40%	Yes
Hung/2012	56	33/20	Western blot	38%	No

^1^Last name of the first author/year of publication.

*SCC* squamous cell carcinoma, *IHC* immunohistochemistry.

Although dysregulation of E2F1 is frequently observed in cancers, it is controversial whether E2F1 participates in oncogenic events to promote tumor growth or functions as a tumor suppressor controlling checkpoint to induce apoptosis [4]. E2F1 overexpression has been shown to be a poor prognostic marker in squamous cell carcinoma of esophagus [21]. However, it has also been demonstrated that increased E2F1 expression correlates with better clinical outcomes in many cancer types such as lymphoma, urinary bladder cancer, tongue cancer, gastric cancer, esophageal adenocarcinoma, colon cancer and breast cancer [11-15,22,23]. In addition to our summary in Table 3 for NSCLC, controversies on the clinical significance of E2F1 expression are noted in breast cancer as well [22,24].

The regulatory mechanism of E2F1 gene expression is complex, and frequently involves both transcriptional and post-transcriptional pathways. The regulation of E2F1 protein levels could be mediated through ubiquitin-proteasome-dependent degradation. UCN-01, a protein kinase C/CDK inhibitor, is a potential anticancer agent. We have previously shown that UCN-01 represses E2F1 expression by promoting proteolysis through a ubiquitin-proteasome-dependent pathway in gastric cancer cells [25]. Translation of E2F1 mRNA can be regulated by microRNA system in cancer [26,27]. Cho and colleagues recently have identified that methylation of E2F1 protein by protein arginine methyltransferase 5 affects the stability of E2F1 protein, and subsequently regulates its function in cell growth and apoptosis [23]. They have shown that E2F1 protein is frequently methylated in cancer cells; decreased level of E2F1 methylation is noted upon DNA damage which stabilizes E2F1, leading to growth inhibition and apoptosis induction. Furthermore, they have found increased levels of protein arginine methyltransferase 5 accompanying with decreased E2F1 protein levels are associated with adverse clinical outcome in colorectal cancer.

Since E2F1 gene expression can be affected at transcriptional and post-transcriptional steps, protein expression probably represents the best way to characterize its biological role in clinical specimen. In this study, we used immunoblotting to quantitate E2F1 protein expression, which could potentially avoid the pitfalls associated with IHC such as staining reaction, operator evaluation, and comparative evaluation. Our report showed that E2F1 protein expression was significantly higher in tumor specimens than normal lung tissue. However, E2F1 overexpression was not a significant prognostic factor for overall survival and probability of freedom from recurrence in our study. Our results are in consistent with the report from Volm et al. [18], but different from other reports [19,20]. The discrepancy of the results among these 4 studies relating to the clinical significance of E2F1 expression in NSCLC (as listed in Table 3) could be due to differences in methodology, sample size, patient population, etc. However, considering all the data available on the clinical significance of E2F1 expression in cancer, it raises a concern for the prognostic role of E2F1 in NSCLC. For future study of E2F1 in NSCLC, a more defined and homogenous patient population such as stage II and III receiving surgery and adjuvant chemotherapy will be preferred. Additionally, incorporating with investigation of proteins regulating E2F1 expression such as protein arginine methyltransferase 5 may provide us more insights.

The strength of the study is that we used immunoblotting to quantitate E2F1 protein expression instead of IHC. There are some limitations of this study that should be mentioned. As a single institute study, the sample size is relatively small. A type I error could therefore have occurred.

## Conclusions

E2F1 protein expression is significantly higher in NSCLC specimens than non-tumor lung tissue. E2F1 overexpression does not adversely impact overall survival and probability of freedom from recurrence.

## Methods

### Patients and tissue procurement

Fifty-six patients with NSCLC who underwent surgical resection in Taipei Veterans General Hospital between January 2001 and June 2003 were enrolled in this study. The tissue procurement protocol was approved by the Institutional Review Board, and written informed consent was obtained from all patients. Fresh tumor specimens and adjacent non-tumor lung tissues were collected in the operating room, snap frozen in liquid nitrogen and stored at −80°C until analysis.

### Protein extraction and Western blot analysis

Protein extraction and Western blot analysis were performed as previously described [28]. Briefly, frozen tissue was homogenized and thawed in ice-cold radioimmunoprecipitation buffer added with 100 μg/ml phenylmethylsulfonyl fluoride, 25 μg/ml Aprotinin, 25 μg/ml Lupeptin, 10 μg/ml soybean trypsin inhibitor, and 1 mM sodium orthovandate. The lysate was incubated on ice for 20 min and then centrifuged at 12,000 rpm for 10 min to sediment the particulate material. Cell lysate containing 50μg of protein from each sample was resolved by SDS-polyacrylamide gel electrophoresis using 8% polyacrylamide. The resolved proteins were transferred onto Immobilon polyvinyl difluoride membranes (Millipore Corporation, Bedford, MA). Ponceau S (Sigma Chemical, St. Louis, MO) staining of the membranes was performed to assess the equivalence of sample loading and gel transfer. Computer densitometry was used to determine the relative loading. The membranes were then destained with tap water for several washes. After blocking with 5% skimmed milk in TBS containing 0.1% Tween 20, the membranes were incubated with rabbit polyclonal antibody against human E2F1 (Santa Cruz Biotechnology, Santa Cruz, CA) as the primary antibody. The blots were then incubated with anti-rabbit horseradish peroxidase-conjugated secondary antibody (Amersham Pharmacia Biotech, Buckinghamshire, UK). The detection of antibody binding was performed by using Pierce SuperSignal Chemiluminescent detection reagents with the protocols recommended by the manufacturer, and blots were exposed to NEN Renaissance X-ray film with intensifying screens. The linear-range signal intensity of E2F1 on the fluorogram was quantitated by a densitometric scanning system. The comparison of E2F1 protein expression was performed after normalization to the densitometric scanning of the Ponceau S staining. The control value of Ponceau S staining in each sample was assigned as an arbitrary densitometry unit (ADU) of 1. The expression of E2F1 was denoted as ADU relative to the corresponding value of Ponceau S staining. Overexpression of E2F1 protein was defined as the ADU ratio of E2F1 in tumor vs. non-tumor exceeding 2.

### Statistical analysis

The overall survival and probability of freedom from recurrence were calculated by the Kaplan-Meier method. The differences of overall survival and probability of freedom from recurrence were compared between groups by log-rank test. To compare between groups with respect to categorical and continuous variables, the *χ*2 test or the independent sample *t*-test was used as appropriate. Statistical analysis was considered to be significant when *P* <0.05.

## Abbreviations

NSCLC, Non-small cell lung cancer; IHC, Immunohistochemistry; ADU, Arbitrary densitometry unit.

## Competing interests

All the authors declare that they have no competing interest.

## Authors’ contributions

CTH and YCW designed the experiments. JJH, KHC and WHH performed the experiments. JJH, CTH and YCW wrote the paper. All authors read and approved the final manuscript.

## References

[B1] Mascaux C Iannino N Martin B Paesmans M Berghmans T Dusart M Haller A Lothaire P Meert AP Noel S The role of RAS oncogene in survival of patients with lung cancer: a systematic review of the literature with meta-analysis Br J Cancer 2005 92 1 131 139 10.1038/sj.bjc.6602258 15597105 PMC2361730

[B2] Steels E Paesmans M Berghmans T Branle F Lemaitre F Mascaux C Meert AP Vallot F Lafitte JJ Sculier JP Role of p53 as a prognostic factor for survival in lung cancer: a systematic review of the literature with a meta-analysis Eur Respir J 2001 18 4 705 719 10.1183/09031936.01.00062201 11716177

[B3] Zheng Z Chen T Li X Haura E Sharma A Bepler G DNA synthesis and repair genes RRM1 and ERCC1 in lung cancer N Engl J Med 2007 356 8 800 808 10.1056/NEJMoa065411 17314339

[B4] Stevens C La Thangue NB The emerging role of E2F-1 in the DNA damage response and checkpoint control DNA Repair (Amst) 2004 3 8–9 1071 1079 15279795 10.1016/j.dnarep.2004.03.034

[B5] Ingram L Munro S Coutts AS La Thangue NB E2F-1 regulation by an unusual DNA damage-responsive DP partner subunit Cell Death Differ 2011 18 1 122 132 10.1038/cdd.2010.70 20559320 PMC3131880

[B6] Van Den Broeck A Nissou D Brambilla E Eymin B Gazzeri S Activation of a Tip60/E2F1/ERCC1 network in human lung adenocarcinoma cells exposed to cisplatin Carcinogenesis 2012 33 2 320 325 10.1093/carcin/bgr292 22159227

[B7] Irwin M Marin MC Phillips AC Seelan RS Smith DI Liu W Flores ER Tsai KY Jacks T Vousden KH Role for the p53 homologue p73 in E2F-1-induced apoptosis Nature 2000 407 6804 645 648 10.1038/35036614 11034215

[B8] Stiewe T Putzer BM Role of the p53-homologue p73 in E2F1-induced apoptosis Nat Genet 2000 26 4 464 469 10.1038/82617 11101847

[B9] Yamasaki L Jacks T Bronson R Goillot E Harlow E Dyson NJ Tumor induction and tissue atrophy in mice lacking E2F-1 Cell 1996 85 4 537 548 10.1016/S0092-8674(00)81254-4 8653789

[B10] Field SJ Tsai FY Kuo F Zubiaga AM Kaelin WG Livingston DM Orkin SH Greenberg ME E2F-1 functions in mice to promote apoptosis and suppress proliferation Cell 1996 85 4 549 561 10.1016/S0092-8674(00)81255-6 8653790

[B11] Moller MB Kania PW Ino Y Gerdes AM Nielsen O Louis DN Skjodt K Pedersen NT Frequent disruption of the RB1 pathway in diffuse large B cell lymphoma: prognostic significance of E2F-1 and p16INK4A Leukemia 2000 14 5 898 904 10.1038/sj.leu.2401761 10803523

[B12] Rabbani F Richon VM Orlow I Lu ML Drobnjak M Dudas M Charytonowicz E Dalbagni G Cordon-Cardo C Prognostic significance of transcription factor E2F-1 in bladder cancer: genotypic and phenotypic characterization J Natl Cancer Inst 1999 91 10 874 881 10.1093/jnci/91.10.874 10340908

[B13] Kwong RA Nguyen TV Bova RJ Kench JG Cole IE Musgrove EA Henshall SM Sutherland RL Overexpression of E2F-1 is associated with increased disease-free survival in squamous cell carcinoma of the anterior tongue Clin Cancer Res 2003 9 10 Pt 1 3705 3711 14506162

[B14] Lee J Park CK Park JO Lim T Park YS Lim HY Lee I Sohn TS Noh JH Heo JS Impact of E2F-1 expression on clinical outcome of gastric adenocarcinoma patients with adjuvant chemoradiation therapy Clin Cancer Res 2008 14 1 82 88 10.1158/1078-0432.CCR-07-0612 18172256

[B15] Evangelou K Kotsinas A Mariolis-Sapsakos T Giannopoulos A Tsantoulis PK Constantinides C Troupis TG Salmas M Kyroudis A Kittas C E2F-1 overexpression correlates with decreased proliferation and better prognosis in adenocarcinomas of Barrett oesophagus J Clin Pathol 2008 61 5 601 605 10.1136/jcp.2007.050963 17908803

[B16] Saiz AD Olvera M Rezk S Florentine BA McCourty A Brynes RK Immunohistochemical expression of cyclin D1, E2F-1, and Ki-67 in benign and malignant thyroid lesions J Pathol 2002 198 2 157 162 10.1002/path.1185 12237874

[B17] Zhang SY Liu SC Al-Saleem LF Holloran D Babb J Guo X Klein-Szanto AJ E2F-1: a proliferative marker of breast neoplasia Cancer Epidemiol Biomarkers Prev 2000 9 4 395 401 10794484

[B18] Volm M Koomagi R Rittgen W Clinical implications of cyclins, cyclin-dependent kinases, RB and E2F1 in squamous-cell lung carcinoma Int J Cancer 1998 79 3 294 299 10.1002/(SICI)1097-0215(19980619)79:3<294::AID-IJC15>3.0.CO;2-8 9645354

[B19] Huang CL Liu D Nakano J Yokomise H Ueno M Kadota K Wada H E2F1 overexpression correlates with thymidylate synthase and survivin gene expressions and tumor proliferation in non small-cell lung cancer Clin Cancer Res 2007 13 23 6938 6946 10.1158/1078-0432.CCR-07-1539 18056168

[B20] Gorgoulis VG Zacharatos P Mariatos G Kotsinas A Bouda M Kletsas D Asimacopoulos PJ Agnantis N Kittas C Papavassiliou AG Transcription factor E2F-1 acts as a growth-promoting factor and is associated with adverse prognosis in non-small cell lung carcinomas J Pathol 2002 198 2 142 156 10.1002/path.1121 12237873

[B21] Yamazaki K Hasegawa M Ohoka I Hanami K Asoh A Nagao T Sugano I Ishida Y Increased E2F-1 expression via tumour cell proliferation and decreased apoptosis are correlated with adverse prognosis in patients with squamous cell carcinoma of the oesophagus J Clin Pathol 2005 58 9 904 910 10.1136/jcp.2004.023127 16126868 PMC1770838

[B22] Kwon MJ Nam ES Cho SJ Park HR Shin HS Park JH Park CH Lee WJ E2F1 expression predicts outcome in Korean women who undergo surgery for breast carcinoma Ann Surg Oncol 2010 17 2 564 571 10.1245/s10434-009-0767-z 19841979

[B23] Cho EC Zheng S Munro S Liu G Carr SM Moehlenbrink J Lu YC Stimson L Khan O Konietzny R Arginine methylation controls growth regulation by E2F-1 EMBO J 2012 31 7 1785 1797 10.1038/emboj.2012.17 22327218 PMC3321197

[B24] Hallett RM Hassell JA E2F1 and KIAA0191 expression predicts breast cancer patient survival BMC Res Notes 2011 4 95 10.1186/1756-0500-4-95 21453498 PMC3078871

[B25] Hsueh CT Wu YC Schwartz GK UCN-01 suppresses E2F-1 mediated by ubiquitin-proteasome-dependent degradation Clin Cancer Res 2001 7 3 669 674 11297263

[B26] Novotny GW Sonne SB Nielsen JE Jonstrup SP Hansen MA Skakkebaek NE Rajpert-De Meyts E Kjems J Leffers H Translational repression of E2F1 mRNA in carcinoma in situ and normal testis correlates with expression of the miR-17-92 cluster Cell Death Differ 2007 14 4 879 882 10.1038/sj.cdd.4402090 17218954

[B27] Yang G Zhang R Chen X Mu Y Ai J Shi C Liu Y Sun L Rainov NG Li H MiR-106a inhibits glioma cell growth by targeting E2F1 independent of p53 status J Mol Med (Berl) 2011 89 10 1037 1050 10.1007/s00109-011-0775-x 21656380

[B28] Wang HW Hsueh CT Lin CF Chou TY Hsu WH Wang LS Wu YC Clinical implications of microsomal prostaglandin e synthase-1 overexpression in human non-small-cell lung cancer Ann Surg Oncol 2006 13 9 1224 1234 10.1245/s10434-006-9001-4 16952028

